# Reactive oxygen species and nitric oxide signaling in bystander cells

**DOI:** 10.1371/journal.pone.0195371

**Published:** 2018-04-05

**Authors:** Kishore Kumar Jella, Roisin Moriarty, Brendan McClean, Hugh J. Byrne, Fiona M. Lyng

**Affiliations:** 1 Department of Radiation Oncology, Emory University, Atlanta, Georgia, United States of America; 2 Radiation and Environmental Science Centre, Focas Institute, Dublin Institute of Technology, Dublin, Ireland; 3 St Luke’s Hospital, Rathgar, Dublin, Ireland; 4 Focas Institute, Dublin Institute of Technology, Dublin, Ireland; 5 School of Physics, Dublin Institute of Technology, Dublin, Ireland; ENEA Centro Ricerche Casaccia, ITALY

## Abstract

It is now well accepted that radiation induced bystander effects can occur in cells exposed to media from irradiated cells. The aim of this study was to follow the bystander cells in real time following addition of media from irradiated cells and to determine the effect of inhibiting these signals. A human keratinocyte cell line, HaCaT cells, was irradiated (0.005, 0.05 and 0.5 Gy) with γ irradiation, conditioned medium was harvested after one hour and added to recipient bystander cells. Reactive oxygen species, nitric oxide, Glutathione levels, caspase activation, cytotoxicity and cell viability was measured after the addition of irradiated cell conditioned media to bystander cells. Reactive oxygen species and nitric oxide levels in bystander cells treated with 0.5Gy ICCM were analysed in real time using time lapse fluorescence microscopy. The levels of reactive oxygen species were also measured in real time after the addition of extracellular signal-regulated kinase and c-Jun amino-terminal kinase pathway inhibitors. ROS and glutathione levels were observed to increase after the addition of irradiated cell conditioned media (0.005, 0.05 and 0.5 Gy ICCM). Caspase activation was found to increase 4 hours after irradiated cell conditioned media treatment (0.005, 0.05 and 0.5 Gy ICCM) and this increase was observed up to 8 hours and there after a reduction in caspase activation was observed. A decrease in cell viability was observed but no major change in cytotoxicity was found in HaCaT cells after treatment with irradiated cell conditioned media (0.005, 0.05 and 0.5 Gy ICCM). This study involved the identification of key signaling molecules such as reactive oxygen species, nitric oxide, glutathione and caspases generated in bystander cells. These results suggest a clear connection between reactive oxygen species and cell survival pathways with persistent production of reactive oxygen species and nitric oxide in bystander cells following exposure to irradiated cell conditioned media.

## Introduction

Radiation induced bystander effects have been observed in unirradiated cells upon receiving signals from irradiated cells [1–6]. The effects include activation of stress inducible signals [7–9], DNA damage [10–13], chromosomal aberrations [14–16], mitochondrial alterations [17], cell death [18–20], changes in gene expression [21, 22] and oncogenic transformation [23].

Bystander signals may be transferred to surrounding cells either by gap junctional intercellular communication or by the production of soluble extracellular factors released from irradiated cells. Soluble signaling factors such as reactive oxygen species (ROS) [24–29], nitric oxide (NO) [28, 30, 31], secondary messengers like calcium [18, 27, 32, 33], cytokines such as interleukins [34–36], transforming growth factor (TGFβ) [29, 37, 38], tumor necrosis factor (TNFα) and (TNF)-related apoptosis-inducing ligand (TRAIL) [39, 40] have been found to play a major role in radiation-induced bystander effects. In recent years, there is increasing evidence suggesting that exosomes play a potential role in transferring signals from irradiated to non-irradiated cells [41–44].

The responses that have been generated by conditioned media indicate that long lived factors can be released by the irradiated cells. It has been reported that conditioned media obtained from irradiated cells could induce intracellular calcium fluxes, increased ROS and loss of mitochondrial membrane permeability in recipient cells [18, 27, 45, 46]. Temme et al reported the release of ROS in non-irradiated cells through TGF-β dependent signaling [47]. The cell membrane could be an important candidate for radiation-induced bystander signaling because an inhibitor of membrane signaling, filipin has been found to suppress bystander effects resulting in the reduction of NO levels [48, 49]. Matsumoto et al revealed that X-irradiation can induce the activation of nitric oxide synthase (iNOS) as early as 3 hours, which resulted in the activation of radioresistance among bystander cells [30]. NO has been found to be one of the key signaling molecules in conditioned media which mediates bystander effects in neoplastic, lymphoma and glioblastoma cells [30, 49, 50]. Ionizing radiation has been found to induce damage to mitochondria with the increase in production of ROS, depolarisation of mitochondrial membrane potential and the release of cytochrome *c* in directly irradiated cells [51]. It was also reported that ICCM can induce changes in mitochondrial distribution, loss of mitochondrial membrane permeability, increase in production of ROS and increase in apoptosis in bystander cells upon receiving conditioned media. These signals were found to be blocked by treatment with antioxidants [18, 52].

Up regulation of MAPK pathway proteins were shown previously in bystander cells [26, 27] and their activation was found to be reduced upon treatment with antioxidants, superoxide dismutase (SOD) and catalase [26]. Previously our group reported the triggering of calcium fluxes and activation of mitogen activate protein kinase (MAPK/MEK) signaling proteins such as extracellular signal-regulated kinase (ERK) and c-Jun N-terminal kinase (JNK) proteins upon addition of conditioned media [27, 46].

Although a number of studies have investigated the role of ROS and NO in bystander cells [18, 46, 53, 54], this study aimed to monitor ROS and NO levels over longer periods in real time after the addition of ICCM and to determine the effect of inhibiting the ERK and JNK pathways on ROS production. Intracellular glutathione levels were also measured after treatment with ICCM to monitor the effect of ROS and NO signals on intracellular antioxidant levels. In addition, caspase activation, cell cytotoxicity and cell viability were measured to determine the mechanism of action of ICCM exposure.

## Materials and methods

### Cell culture

Human keratinocyte cells (HaCaT cells) (Cell Lines Services, Germany) were cultured in Dulbecco’s Modified Eagle Medium (DMEM): F-12 Ham (1:1) (Sigma Aldrich, Dorset, UK) containing 10% fetal bovine serum (FBS), 5000 IU/ml of penicillin streptomycin (Gibco Biocult, Irvine, UK) solution and 1 μg/ml of hydrocortisone (Gibco Biocult). The cells were maintained in an incubator at 37°C, with 95% humidity and 5.0% CO_2_. Subculture was routinely performed when cells were 75–80% confluent using 0.25% trypsin (Sigma Aldrich) and 1 mM versene (Sigma Aldrich) at a 1:1 ratio.

### Irradiation

Culture flasks (T-25 flasks, Sarstedt, Wexford, Ireland) were sealed and irradiated (0.005, 0.05, 0.5 Gy) using a cobalt-60 teletherapy source at St. Luke’s hospital, Dublin. For 0.5 Gy dose point the source to sample distance was 80 cm, for 0.05 Gy and 0.005 Gy the source to sample distance is 191.5 cm. The dose rate delivering approximately 1.5 Gy/min during these experiments. Thermoluminescent dosimeters (TLD) were used to confirm that the appropriate dose was delivered. Control flasks (0 Gy) were removed from the incubator and handled under the same conditions as the irradiated cells. The flasks were placed in the incubator immediately after irradiation.

### Harvesting of ICCM

Donor flasks containing 200,000 cells were seeded and irradiated 6 hours after plating. Medium from both irradiated and unirradiated cells were poured off donor flasks after 1 hour of irradiation and filtered through a 0.22 μm filter (Nalgene/Thermo Fisher, Hereford, UK) to prevent debris and cells transferring into the medium. After filtration, the medium was aliquoted and stored at -80°C.

### Time lapse imaging setup

Approximately 7 X 10^4^ cells were grown on 35 mm glass bottom culture dishes (Mat Tek Corporation, Ashland, MA, USA; # P35G-0-20-C). After 24 hours of plating, cells were washed twice with PBS and loaded with 2.5 μM 5’, 6’-chloromethyl-2’, 7’-dichlorodihydrofluorescein diacetate (CM-H2DCFDA dye) (Invitrogen/Molecular Probes, Invitrogen, Paisley, UK) for ROS and 5 μM 4-amino-5-methylamino-2’,7’-difluorofluorescein diacetate (DAF-AM dye) (Invitrogen/Molecular Probes) for NO using DMEM: F-12 Ham media without phenol red (Sigma Aldrich). After 30 mins incubation at 37°C with the appropriate dye the cells were washed thrice with PBS and treated with 0 Gy and 0.5 Gy ICCM. Cells were imaged using Axiovert 200M inverted microscope (Carl Zeiss, Welwyn Garden City, Hertfordshire, UK). The microscope is equipped with Plan-APOCHROMAT 63 X / NA1.4 oil immersion lens, and an incubation chamber mounted on the microscope to maintain stable temperature of 37°C and 5% CO2. The image acquisition and interactive measurement was performed using Axiovision rel 4.8 software. Live cell imaging was performed using bright field and FITC fluorescence mode. The fluorescent dyes were excited using Axiovert HBO lamp (100 W). Hydrogen peroxide (H_2_O_2_) was used as positive control to identify the ROS production.

### Image acquisition

To analyse the fluorescent signals, HaCaT cells were treated with 0 Gy and 0.5 Gy ICCM and monitored for approx. 24 hours for ROS and 6 hours for NO with an interval of 30 mins. The exposure time for the dyes was kept to a minimum and the power of HBO lamp was attenuated to a minimal level by using neutral density filters transmitting 20% of incident light. To control the effects of phototoxicity, cells treated with 0 Gy ICCM were monitored for the same time as 0.5 Gy treated cells using the same fluorescent dyes and time intervals. The fluorescence was recorded at 480 nm excitation/520 emission.

### Intracellular measurement of ROS and glutathione (GSH) levels

Intracellular ROS and GSH levels were measured in HaCaT cells using CM-H2DCFDA dye (Invitrogen/Molecular probes) and ThiolTracker^TM^ Violet (Invitrogen/Molecular Probes) respectively upon treatment with 0.005 Gy, 0.05 Gy and 0.5 Gy ICCM. For both the assays, the study was performed in black 96-well microplates (Nunc, Roskilde, Denmark) and the cells were seeded at the density of 1x10^5^ cells/ml in 100 μl of respective media. After 24 hours of plating, the cells were loaded with 2.5 μM CM-H2DCFDA dye and 20 μM ThiolTracker^TM^ Violet for 30 mins at 37 ^ο^C for both the assays separately. After incubation with dyes, plates were washed with PBS three times before treatment with 0 Gy, 0.005 Gy, 0.05 Gy and 0.5 Gy ICCM and left at 37°C, 5% CO_2_ incubator. The fluorescence intensities were measured after 5 min, 30 min, 1 hour, 1.5, 2, 2.5, 5, 10, 15, 20 and 24 hours respectively. For ROS assay, a Tecan microplate reader (TECAN GENios, Grodig, Austria) was used with excitation and emission wavelengths set at 485 nm and 530 nm. For Glutathione assay, a VICTOR3V™1420 Multilabel Counter plate reader (Perkin Elmer, Buckinghamshire, UK) was used with excitation and emission wavelengths set at 405 nm and 535 nm.

### ApoTox-Glo triplex assay

The assays were performed using a kit obtained from Promega Corporation (Madison, Wisconsin, USA), which enables cell viability, cytotoxicity and caspase activation within a single assay well in 96-well plate. Approximately 1X10^5^ cells/ml were plated in clear-bottom 96-well plates. After 24 hours of incubation, the cells were treated with 0 Gy, 0.005 Gy, 0.05 Gy and 0.5 Gy ICCM and incubated for 2, 4, 6, 8, 12, 24, 48 hours. 20 μl of viability and cytotoxicity reagent containing both glycyl phenylalanyl-aminofluorocoumarin (GF-AFC) substrate and bis-alanylalanyl-phenylalanyl-rhodamine 110 (bis-AAF-R) 110 substrate was added to respective wells and the contents were mixed briefly using an orbital shaker at 240 rpm for one minute. The substrate GF-AFC can enter into live cells where the cell membrane integrity is active and it is cleaved by live-cell protease to release AFC as byproduct. The other substrate, bis-AAF-R110 cannot enter into live cells but can be cleaved by dead-cell protease released from cells that have lost membrane integrity to produce R110. The cells were incubated for 30 min at 37°C and the fluorescence signals were recorded at 400_EX_/505_EM_ to measure cell viability and 485_EX_/520_EM_ to measure cytotoxicity. After measuring both cell cytotoxicity and viability, 100 μl of the caspase-Glo 3/7 was added to the respective wells to measure the signal generated by luminescence due to caspase activation. The luminescence was measured after 30 mins of incubation using a Tecan microplate reader. To check cell viability, cell cytoxicity and caspase activation, the drug campothecin was used as positive control.

### MAPK inhibitors

HaCaT cells were exposed to MEK and JNK inhibitors and treated with ICCM and ROS levels were monitored. The cells were loaded with 2.5 μM CM-H2DCFDA dye for 30 mins and they were incubated with PD98059 (MEK inhibitor) at a concentration of 20 μM and SP600125 (JNK inhibitor) at a concentration of 10 μM for 15 min before the addition of 0.5 Gy and 0 Gy ICCM [27].

### Statistical analysis

The statistical analyses were performed using Statgraphics Centurion XV software (StatPoint Technologies, Inc., Warrenton, Virginia, USA) and GraphPad prism software (Version 7, La Jolla, USA). Statistical differences between multiple comparisons were calculated using one-way analysis of variance (ANOVA), a p-value of < 0.05 was considered to be statistically significant.

## Results

### ROS signaling using time lapse imaging

**Fig 1:**
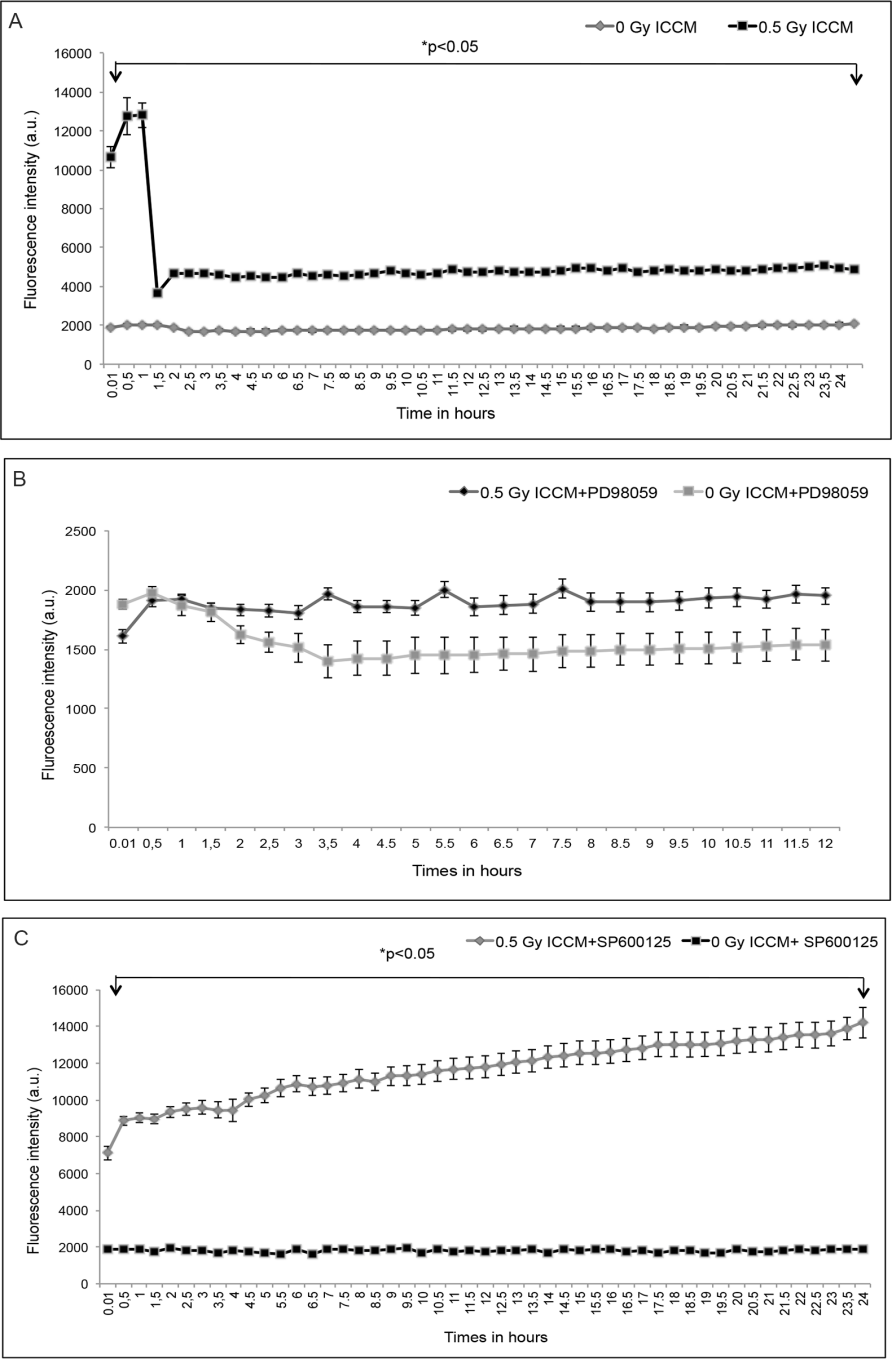
ROS signaling in bystander cells. (A) ROS time lapse fluorescence intensity. (B) Inhibition of ROS after the addition of 0.5 Gy ICCM along with ERK inhibitor (PD98059). (C) Increase in production of ROS after the addition of 0.5 Gy ICCM in combination with JNK inhibitor (SP600125).

ROS staining was performed using a fluorescent dye CMH2DCFDA dye. The fluorescence intensity (Fig 1A) was measured within 1 min after the addition of 0.5 Gy ICCM and was continuously monitored for approx. 24 hours with an interval of 30 mins. An increase in ROS levels was found immediately after the addition of 0.5 Gy ICCM, which further increased after 30 mins and was maintained up to 1 hour. After 1 hour, a decrease in ROS levels was observed and thereafter a constant production of ROS was observed up to 24 hours. There was no increase in ROS production after the addition of 0 Gy ICCM (Fig 1A). To elucidate the relationship between ROS and the MAPK pathway, inhibitors of MEK and JNK were used. Fig 1B shows the inhibition of ROS after the addition of an ERK inhibitor (PD98059) and the results were similar to those for 0 Gy ICCM with no ROS production observed. The cells were able to survive only for a period of 12 hours and underwent cell death on further incubation. The cells were also incubated with a JNK inhibitor (SP600125) before the addition of 0.5 Gy ICCM and these cells showed a significant increase in ROS production (Fig 1C) compared with 0 Gy ICCM. After the addition of JNK inhibitor and 0.5 Gy ICCM, the cells were able to survive for 24 hours without undergoing cell death.

### NO signaling using time lapse imaging

**Fig 2:**
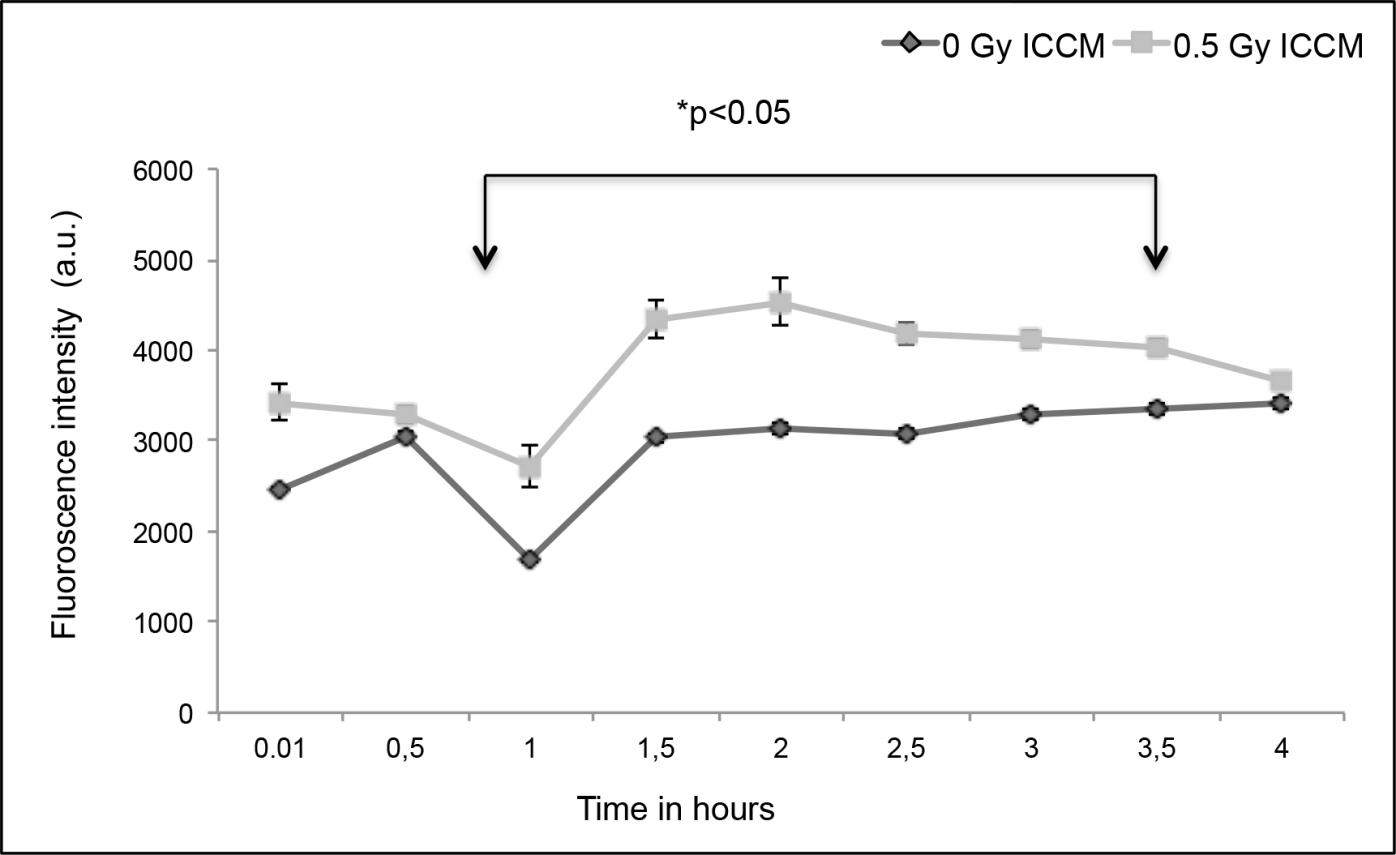
NO signaling in bystander cells. NO signaling in HaCaT cells after the addition of 0.5 Gy ICCM and 0 Gy ICCM.

NO staining was performed using a fluorescent dye DAF-FM. The fluorescence intensities were measured in HaCaT cells after loading with DAF-AM dye and levels of NO were measured after the addition of 0.5 Gy ICCM (Fig 2). One hour after the addition of ICCM, a significant increase in production of NO was observed and maintained up to 3.5 hours. After 4 hours, the fluorescence returned to control levels. There was no increase in NO production after the addition of 0 Gy ICCM.

### Intracellular measurement of ROS and glutathione level

**Fig 3:**
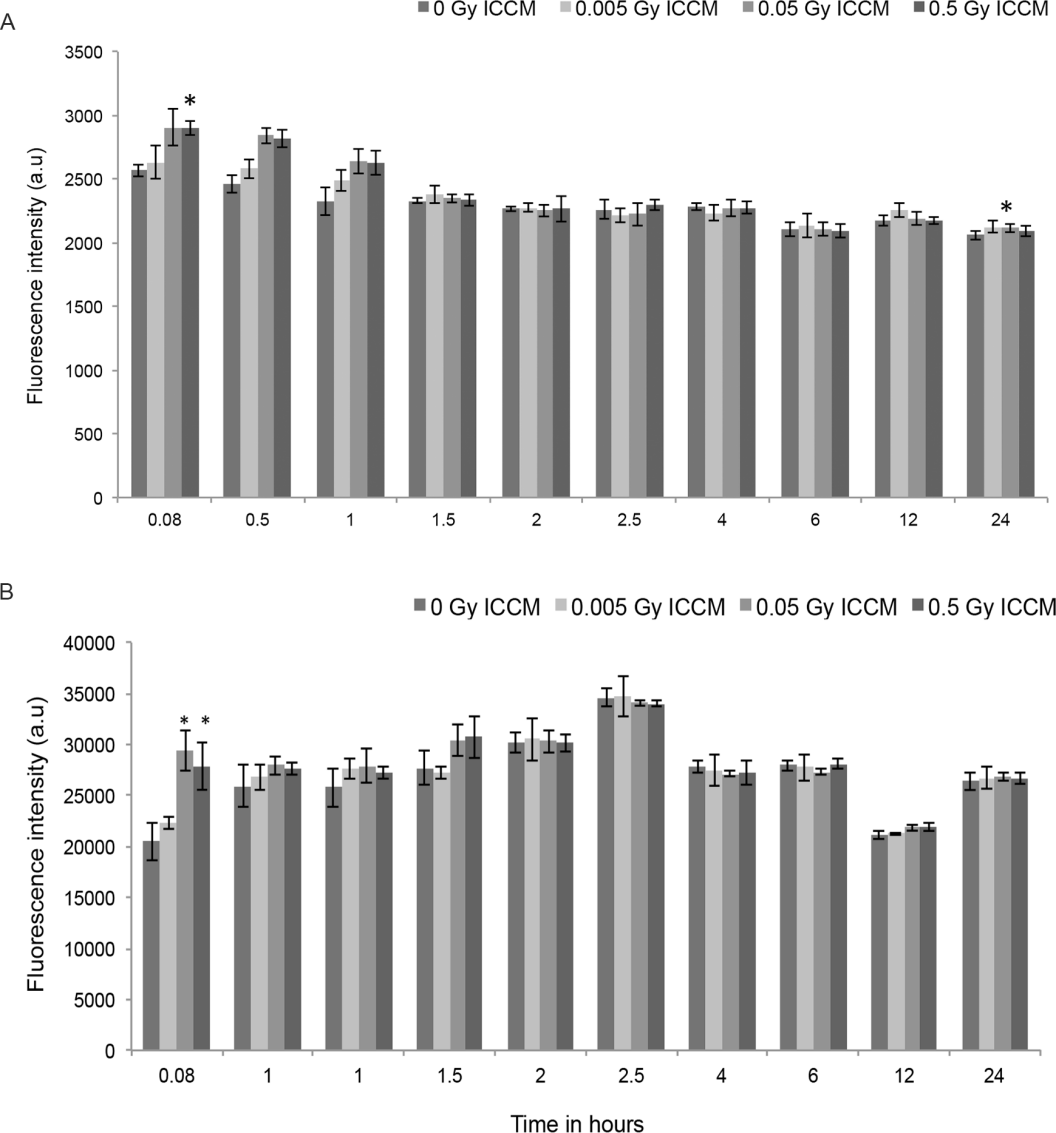
Fluorescence intensity levels for ROS and GSH were measured in bystander cells after the addition of 0.005 Gy, 0.05 Gy and 0.5 Gy ICCM. (A) Fluorescence intensity levels for ROS using CM-H2DCFDA dye in HaCaT cells after the addition of conditioned media. (B) Fluorescence intensity levels for Glutathione using ThiolTracker^TM^ Violet in HaCaT cells after the addition of conditioned media.

The intracellular fluorescence intensity levels for ROS was measured using CM-H2DCFDA dye, after the addition of 0 Gy, 0.005 Gy, 0.05 Gy and 0.5 Gy ICCM in the global cell population using a plate reader assay. The fluorescence intensity was measured after 5 mins, 0.5, 1, 1.5, 2, 2.5, 4, 6, 12 and 24 hours respectively as shown in Fig 3A. There was a significant increase in fluorescence intensity in cells after adding 0.05 Gy and 0.5 Gy ICCM and this increase was maintained up to one hour. After one hour, the fluorescence intensity levels were similar to those in cells treated with 0 Gy ICCM. To assess intracellular redox state, fluorescence intensity levels for cellular GSH was measured using ThiolTracker^TM^ Violet. Fluorescence intensity levels of GSH was measured in HaCaT cells after 5 mins, 0.5, 1, 1.5, 2, 2.5, 4, 6, 12 and 24 hours treatment with 0.005 Gy, 0.05 Gy and 0.5 Gy ICCM and compared with 0 Gy ICCM treated cells as shown in Fig 3B. A significant increase in fluorescence intensity levels was observed 5 mins after the addition of 0.05 Gy and 0.5 Gy ICCM. In the first half an hour, a significant increase in fluorescence intensity levels were observed up to 1 hour in cells treated with 0.005 Gy, 0.05 Gy and 0.5 Gy ICCM and the increase in fluorescence intensity levels was slightly higher in 0.05 Gy ICCM treated cells when compared with 0.5 Gy ICCM. At one and a half hours, a small but not significant increase in fluorescence intensity levels were maintained in cells treated with 0.05 Gy and 0.5 Gy ICCM but not in 0.005 Gy ICCM. After one and a half hours, the fluorescence intensity levels in cells treated with 0.005 Gy, 0.05 Gy and 0.5 Gy ICCM were similar to the control level (0 Gy ICCM) and this level was maintained up to 24 hours.

### ApoTox-triplex assay

**Fig 4:**
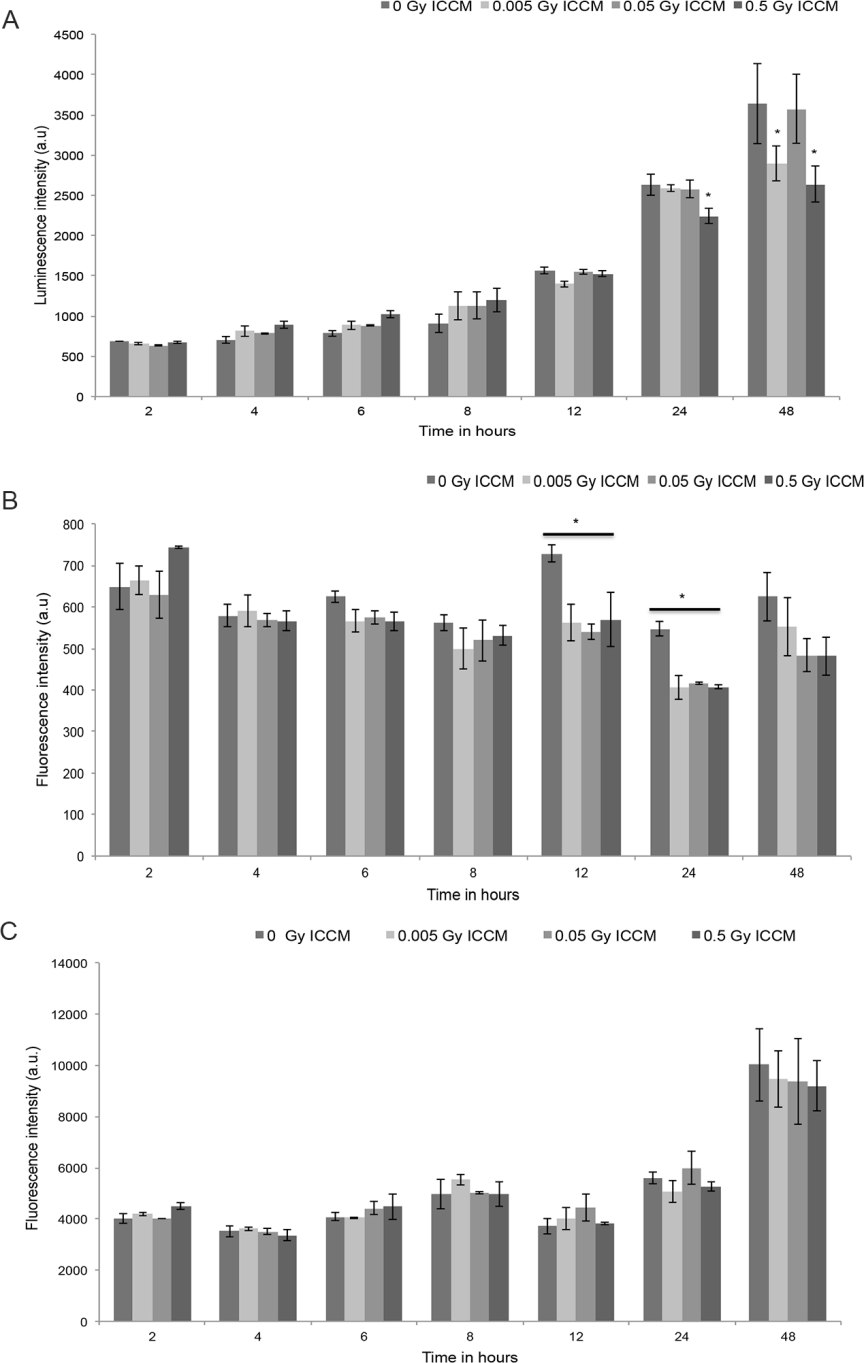
Luminescence levels indicating caspase activation (A) and fluorescence intensity levels indicating cell viability (B) and cytotoxicity (C) in bystander cells after the addition of 0.005 Gy, 0.05 Gy and 0.5 Gy ICCM.

In this assay, the fluorescence intensity levels were measured for cell viability and cytotoxicity and caspase activation was measured using luminescence assay. Fluorescence intensity levels for cell viability, cytoxicity and luminescence for caspase activation was measured at 2, 4, 6, 8, 12, 24 and 48 hours after treatment with 0.005 Gy, 0.05 Gy and 0.5 Gy ICCM and compared with 0 Gy ICCM (Fig 4). A slow increase in luminescence indicating caspase activation (Fig 4A) was found up to 8 hours of treatment with ICCM. A significant decrease in caspase activation was found in 0. 5 Gy ICCM treated cells after 24 and 48 hours. Fluorescence intensity levels indicating cell viability (Fig 4B) was found to decrease up to 8 hours and after 12, 24 hours, there is a significant decrease in cell viability in HaCaT cells treated with 0.005 Gy, 0.05 Gy and 0.5 Gy ICCM when compared with 0 Gy treated cells. A small but not significant increase in fluorescence intensity levels indicating cytotoxicity was observed at 2 hours after exposure for 0.5 Gy ICCM, at 8 hours for 0.005 Gy and 0.05 Gy ICCM and at 12 hours for 0.005 Gy ICCM (Fig 4C). A small but not significant decrease in fluorescence intensity levels was observed at 24 hours for 0.005 Gy and 0.5 Gy ICCM and at 48 hours for 0.05 Gy and 0.5 Gy ICCM (Fig 4C). Statistical analysis between the time points of caspase activation, cell viability, and cell cytotoxicity was shown in Tables 1–3.

In the below Tables, * represent p<0.05, ** represent p<0.01, *** represent p<0.0001.

**Table 1:** Viability.

Dose point (Gy)	Comparison between hours	Significance
0.005 Gy	2 & 6	*
	2 & 24	***
	4 & 24	*
0.05 Gy	2 & 24	***
0.5 Gy	2 & 4	*
	2 & 6	*
	2 & 8	****
	2 & 24	***

**Table 2:** Cytotoxicity.

Dose point (Gy)	Comparison between hours	Significance
0.005 Gy	2 & 48	**
	4 & 48	**
	6 & 48	**
	12 & 48	**
	24 & 48	*
0.05 Gy	2 & 48	**
	4 & 48	**
	6 & 48	**
	8 & 48	*
	12 & 48	**
0.5 Gy	2 & 48	*
	4 & 48	**
	6 & 48	*
	8 & 48	*
	12 & 48	**

**Table 3:** Caspase activation.

Dose point (Gy)	Comparison between hours	Significance
0.005	2 & 24	***
	2 & 48	****
	4 & 48	***
	4 & 24	***
	4 & 48	***
	6 & 24	**
	6 & 48	***
	8 & 24	**
	8 & 48	**
	12 & 24	*
	12 & 48	**
0.05	2 & 24	***
	2 & 48	****
	4 & 24	***
	4 & 48	****
	6 & 24	**
	8 & 24	**
	8 & 48	***
0.5 Gy	2 & 24	**
	2 & 48	***
	4 & 48	**
	6 & 24	*
	6 & 48	**
	8 & 48	**

## Discussion

This study showed the production of intercellular signaling molecules such as ROS for 24 hours and NO for 4 hours after the addition of ICCM. Inhibition of the ERK pathway appeared to inhibit ROS production whereas inhibition of the JNK pathway appeared to increase ROS production over time. ROS and GSH levels were also found to be increased after treatment in the total cell population using a microplate reader assay. The ApoTox-Glo triplex assay showed an increase in caspase activation up to 8 hours, decrease in cell viability up to 48 hours and no significant change in cell cytotoxicity after the addition of ICCM.

Previously, there have been a number of studies on bystander cells studying membrane signaling [48, 49, 55, 56]. Simultaneous study of membrane and calcium signaling revealed that membrane signaling is an initial event produced in bystander cells soon after the addition of ICCM [57]. It was reported that mitochondrial calcium uptake is involved in the production of ROS in irradiated cells [58]. The cellular stress produced by radiation may persist for a longer time due to its effects on oxidative metabolism [59]. Plasma membrane bound NADPH oxidase might play a role for production of ROS for longer times in bystander cells [26, 60]. The activation of NADPH oxidase mainly results in the formation of superoxide anion as a major product along with hydrogen peroxide, the hydroxyl radical and hypochlorous acid as other by-products [61]. ROS production in mitochondria was also reported in bystander cells [62]. Increased mitochondrial mass was also observed with the increase in production of ROS which further reduced the function of mitochondria [63]. In our present study, the production of ROS was monitored for a period of 24 hours with an interval of 30 mins in real time using time lapse microscopy. To confirm continuous production of ROS, a microplate reader assay was also performed to detect global ROS production in HaCaT cells after the addition of ICCM. This method has allowed us to identify the production of ROS for one hour, however after that no increase in ROS production was observed and this may be due to the measurement of ROS production from the total cell population whereas in time lapse microscopy, ROS production was measured in single cells. The continuous generation of ROS could be due to signals present in ICCM that have been generated after irradiation. The de novo generation of ROS could result in the activation of the nuclear factor kappa B (NF-kB) pathway resulting in cell survival and proliferation by inhibiting cell death [9]. Previously our group identified reduction of MMP at 6 hours and hyperpolarization of MMP after 18 hours in the HPV-G cell line after treatment with ICCM [8]. X-ray irradiation of AGO1522 cell line, induced an increase in ROS levels which results in DNA damage in bystander cells [64]. Ionizing radiation in mouse models has led to a decrease in DNA damage in the presence of ROS scavengers which is further followed by an increase in cell survival and decreased occurrence of apoptosis [65, 66]. Elevated levels of ROS in bystander HepG2 cells resulted in micronucleus formation and autophagy upon exposure to conditioned media obtained after irradiation [67]. SirT1 was found to play a role in increased ROS accumulation in bystander hepatocyte cells that could play a protective role in bystander effects [68]. The role of NO in bystander responses has been identified by different groups [28, 30, 31, 38]. In this study, we identified the slow increase in NO levels in bystander cells after treatment with conditioned media. This finding supports our previous data showing a subsequent increase in NO levels after the addition of ICCM in HaCaT cells [57]. NO was identified to be involved in bystander effects in fibroblast cells, the same group identified the role of transforming growth factor (TGF-β) in NO production [69]. It was also reported that TGF- β was an important factor involved in bystander signaling which plays an important role to raise the level of ROS and NO and further cause DNA damage [38]. Oxygen free radicals and NO produced from mitochondria were found to initiate and activate the early process of radiation induced bystander effects [58]. NO dependent DNA breaks were observed in bystander cells on exposure to low dose alpha particle radiation, the authors suggested that this early bystander effect could be cNOS dependent [54]. In our present study, we identified the continuous production of ROS and increased glutathione levels in HaCaT cells. In Hsp27 transfected Jurkat cells, the protein Hsp27 was found to play a major role in protecting the cells against radiation induced apoptosis, underlying signaling mechanism resulting to radiation resistance which further involves in production of ROS and increase in glutathione levels [70]. In this study, caspase 3/7 activation has been found to increase 4 hours after the addition of ICCM and this increase was maintained up to 8 hours and then reduced up to 24 hours. Increased expression of Bcl-2 proteins has also observed by our group in HPV-G cells upon exposure to ICCM [52]. Low levels of apoptosis were also observed in HaCaT cells after treatment with ICCM [71]. Furlong et al demonstrated the initiation of an apoptotic signaling pathway in bystander cells with activation of various caspases [72].

Studies have shown that the MAPK pathway is activated by the production of ROS [73]. Various cellular stimuli that induce the production of ROS also resulted in the activation of MAPK pathway in many cell types [73, 74]. Activated forms of ERK, JNK were found after the addition of ICCM in HPV-G treated cells [27]. Activation of the JNK and p38 MAPK pathways were found to be involved in activation of pro-apoptotic responses, whereas activation of the ERK pathway was found to be involved in anti-apoptotic responses [75]. In our study, inhibition of ERK activation (PD98059) was found to abrogate the production of ROS in ICCM treated cells and caused cells to undergo cell death after 12 hours and this might be due the inhibition of anti-apoptotic signals within the cells. The inhibitor PD98059 was found to partially inhibit H_2_O_2_ induced decrease in mitochondrial membrane permeability in cultured renal proximal tubular cells (RPTC) and partially blocked mitochondrial swelling in isolated renal cortical mitochondria (RCM) [76]. Inhibition of the JNK pathway (SP600125) resulted in an increase in the production of ROS by inhibiting apoptosis signals which is a well known effect of JNK [75]. The inhibitor SP600125 was found to increase in mitochondrial membrane permeability (MMP) and it resulted in the accumulation of ROS in RN22 schwannoma cells [77].

Previously our group reported 20–30% clonogenic cell death in HaCaT cells at 0.05 Gy and 0.5 Gy but not at 0.005 Gy ICCM [46]. A reduction in clonogenic survival was also observed in the HPV-G cell line following exposure to 0.005 Gy ICCM [78, 79]. A significant decrease in cell viability was observed after the addition of 0.05 Gy, 0.5 Gy ICCM after 24 hours [46]. In our present study, reduction in cell viability was observed for 24 hours after treatment with ICCM in bystander cells. In most cases, both viability and cytotoxicity are inversely correlated. But in our study, no significant change in cytotoxicity was observed up to 24 hours. In a previous study, we observed cell cycle disturbances after the addition of ICCM in HaCaT cells [71]. A reduction in cell viability without a concomitant increase in cell cytotoxicity was observed due to disturbances in cell cycle phases and alters the cell division without inducing any changes in its membrane integrity [80].

In summary, this study has identified signaling molecules such as ROS and NO in real time after the addition of ICCM. Inhibitors of MAPK pathway were used to modulate ROS production in bystander cells. The ApoTox-Glo triplex assay has been performed after the addition of ICCM and showed an increase in caspase activation up to 8 hours and reduction up to 48 hours. A decrease in cell viability with no significant changes in cell cytotoxicity may be due to initial cell cycle disturbances reported previously [71]. Our study has identified a clear connection between ROS and MAPK pathway in bystander cells.

## Supporting information

S1 File ROS signaling in bystander cells. It contains the data sets of Fig 1A–1C as S1A-C.
(XLSX)

S2 File NO Signaling in bystander cells. It contains the data of Fig 2.
(XLSX)

S3 File Intracellular measurements of ROS and GSH levels. The file contains the data sets for Fig 3A and 3B as S3A and S3B.
(XLSX)

S4 File Apo-Tox triplex assay. It contains the data sets of viability assay, caspase activation and cytotoxicity assay data sets.
(XLSX)
